# SCIMP is a transmembrane non-TIR TLR adaptor that promotes proinflammatory cytokine production from macrophages

**DOI:** 10.1038/ncomms14133

**Published:** 2017-01-18

**Authors:** Lin Luo, Nilesh J. Bokil, Adam A. Wall, Ronan Kapetanovic, Natalie M. Lansdaal, Faustine Marceline, Belinda J. Burgess, Samuel J. Tong, Zhong Guo, Kirill Alexandrov, Ian L. Ross, Margaret L. Hibbs, Jennifer L. Stow, Matthew J. Sweet

**Affiliations:** 1 Institute for Molecular Bioscience (IMB), The University of Queensland, Brisbane, Queensland 4072, Australia; 2 IMB Centre for Inflammation and Disease Research, The University of Queensland, Brisbane, Queensland 4072, Australia; 3 Department of Immunology and Pathology, Alfred Medical Research and Education Precinct, Monash University, Commercial Road, Melbourne, Victoria 3004, Australia

## Abstract

Danger signals activate Toll-like receptors (TLRs), thereby initiating inflammatory responses. Canonical TLR signalling, via Toll/Interleukin-1 receptor domain (TIR)-containing adaptors and proinflammatory transcription factors such as NF-κB, occurs in many cell types; however, additional mechanisms are required for specificity of inflammatory responses in innate immune cells. Here we show that SCIMP, an immune-restricted, transmembrane adaptor protein (TRAP), promotes selective proinflammatory cytokine responses by direct modulation of TLR4. SCIMP is a non-TIR-containing adaptor, binding directly to the TLR4-TIR domain in response to lipopolysaccharide. In macrophages, SCIMP is constitutively associated with the Lyn tyrosine kinase, is required for tyrosine phosphorylation of TLR4, and facilitates TLR-inducible production of the proinflammatory cytokines IL-6 and IL-12p40. Point mutations in SCIMP abrogating TLR4 binding also prevent SCIMP-mediated cytokine production. SCIMP is, therefore, an immune-specific TLR adaptor that shapes host defence and inflammation.

## Abstract (web summary)

Toll-like receptors engage TIR domain-containing adaptors to control proinflammatory gene expression in response to pathogens and tissue damage. Here the authors show that the non-TIR domain-containing transmembrane protein SCIMP is a previously unrecognized TLR adaptor expressed by macrophages.

Cells of the innate immune system such as macrophages use several families of pattern recognition receptors to detect and respond to danger signals presented during infection, injury and/or perturbed homeostasis^1^,^2^. The Toll-like receptors (TLRs), single-pass transmembrane proteins at the cell surface and in endolysosomal compartments, are the most extensively studied of the pattern recognition receptors. Activation of TLRs by pathogen-derived or host-derived stimuli triggers proinflammatory signalling cascades, leading to the production of proinflammatory mediators that are required for effective host defence, inflammation and homeostatic repair processes. TLR4, the archetypal member of the TLR family, cooperates with CD14 and MD2 to instigate responses to lipopolysaccharide (LPS) from Gram-negative bacteria^3^. Ligand-bound, dimerized TLR4 undergoes homotypic interactions with the intracellular Toll/interleukin-1 receptor (TIR) domains of the TIR-containing adaptors MAL and TRAM to initiate MyD88-dependent and TRIF-dependent TLR signalling, respectively^4^,^5^,^6^. Spatiotemporal binding of TLR4 to different adaptors at the cell surface or in endosomal compartments generates distinct signalling and transcriptional programs to provide specificity to inflammatory mediator outputs^7^,^8^,^9^,^10^. Other members of the TLR family also couple with TIR-containing adaptors at the cell surface or in endosomes to generate specificity in signalling responses. The nature of the specific cytokines produced on TLR activation is critical for mounting a robust and effective immune defence, and for the subsequent resolution of inflammation and restoration of homeostasis. However, TIR-containing adaptor proteins are widely expressed, and additional mechanisms or adaptors that enable either heightened or more selective inflammatory responses in innate immune cells are not well understood.

Members of the transmembrane adaptor protein (TRAP) family scaffold signalling proteins and kinases to support receptor-mediated signalling in other immune cells^11^. For instance, linker of activation for T cells (LAT) directly and indirectly recruits and activates signalling molecules, including Grb2, PLCγ1, SLP-76 and Vav1, downstream of activated T-cell receptors^12^. Consequently, LAT is essential for T cell development^13^,^14^, and for mature T cell functions^15^. LAT is a member of the palmitoylated TRAP (pTRAP) subfamily, which is notable for its signature palmitoylation-directed targeting to specific membrane microdomains^11^. Current evidence for pTRAP regulation of innate immune receptors is limited, resting on a small number of studies relating to TREM signalling in macrophages^16^,^17^. To date, specific roles for TRAPs in direct TLR signalling have not been investigated.

The most recently described member of the pTRAP family is SLP adaptor and C-terminal Src kinase (CSK)-interacting membrane protein (SCIMP), which has been reported to associate with Src family kinases (SFKs) and to regulate MHC class II signalling in B cells^18^ and Dectin-1 signalling in dendritic cells^19^. Recent evidence also posits *SCIMP* as a potential disease-related gene in human autoimmune and neurodegenerative conditions^20^,^21^. In this study, we examine possible roles for pTRAPs in TLR-driven innate immune responses. We find that SCIMP acts as an unconventional, non-TIR-containing TLR adaptor in macrophages, directly binding to TLR4 and orchestrating its ligand-induced tyrosine phosphorylation. Moreover, we show that SCIMP is a TLR signalling adaptor that provides remarkable selectivity to proinflammatory cytokine responses downstream of multiple TLRs. SCIMP is thus an important regulator of host innate immune responses to danger signals.

## Results

### SCIMP is a transmembrane adaptor for TLR4 in macrophages

**Figure 1:**
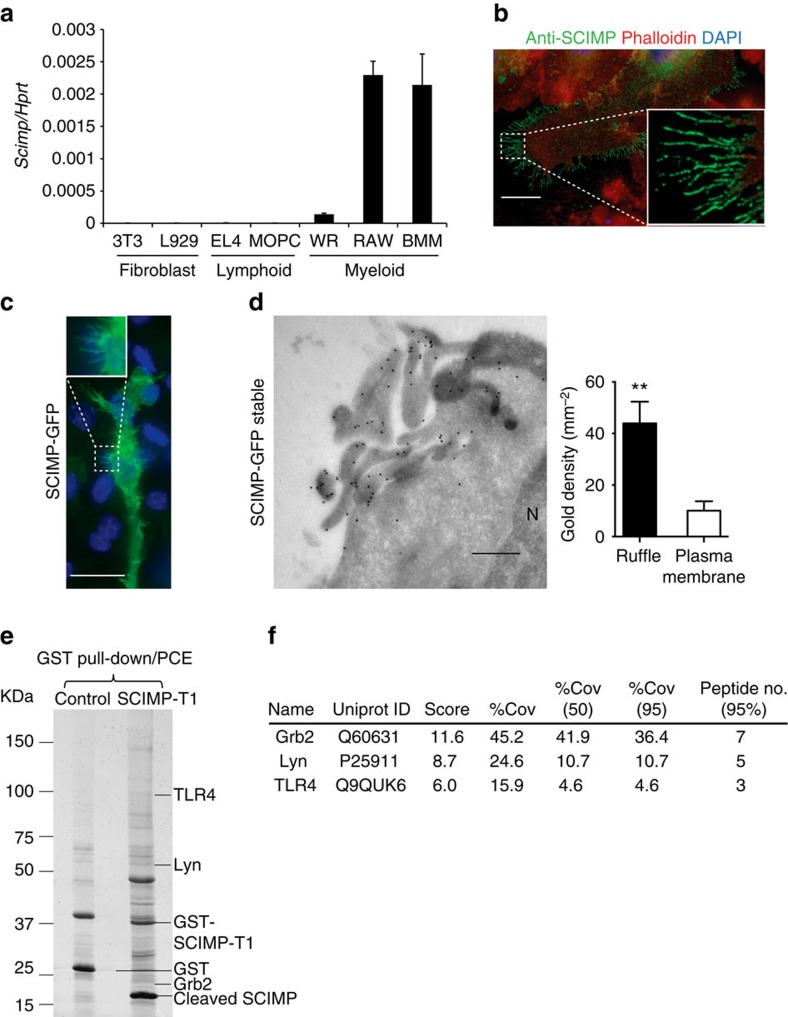
Immune-restricted SCIMP is a TLR4-associated cell surface protein enriched in microdomains. (**a**) mRNA expression of SCIMP was assessed in the indicated fibroblast, lymphoid and myeloid cell lines. Data represents mean+s.e.m. (*n*=3). (**b**) Immunostaining of endogenous SCIMP in LPS-activated RAW264.7 cells (green) on filopodia; cells were co-stained with phalloidin (red) and 4′,6-Diamidino-2′-phenylindole dihydrochloride (DAPI; blue). (**c**) Fluorescent imaging of LPS-treated (30 min) RAW264.7 cells transiently transfected with SCIMP-GFP (green). The cells were co-stained with DAPI (blue). (**d**) Immunogold labelling on cryo-EM sections of RAW264.7 cells stably expressing GFP-SCIMP. GFP labelling ruffles at the cell surface. N=nucleus. Gold particles on ruffle or filopodia membranes versus other stretches of plasma membrane were counted (*n*=5 cells). Significance was assessed using the Student's *t*-test (**P<0.01). (**e**) GST-SCIMP-T1 coupled to GSH-Sepharose was used for pull-downs from LPS-activated RAW264.7 cell extracts. Bound proteins were eluted by a protease cleavage elution method and separated by SDS–PAGE. Excised bands were identified by liquid chromatography and mass spectrometry (LC/MS/MS). A band at ∼100 kDa, absent from the GST control, was identified as TLR4. (**f**) List of the top hits from the LC/MS/MS analysis of SCIMP-GST pull downs. Data in **a**–**f** are representative of at least three independent experiments. Scale bars in **b**–**d** represent 10 μm, 20 μm and 10 nm, respectively.

Examination of pTRAP family member expression across immune and non-immune cell populations highlighted the marked expression of SCIMP in primary mouse bone marrow-derived macrophages (BMM), as well as in the RAW264.7 and WR19M macrophage-like cell lines, whereas it was not detectable in the non-myeloid cells that were screened (Fig. 1a). No other pTRAP displays this selective expression pattern (Supplementary Fig. 1a), and we were drawn to further investigate possible SCIMP functions in macrophages. The cellular localization of SCIMP was examined by immunostaining in primary macrophages (BMM; Supplementary Fig. 1b) and in RAW264.7 cells (Fig. 1b). In both cases, SCIMP staining was apparent on intracellular punctate membranes, but was particularly prominent on the macrophage plasma membrane, where it was further concentrated in cell surface projections, notably the filopodia and ruffles of some cells. This localization was replicated by expression of green fluorescent protein (GFP)-SCIMP (Fig. 1c), and at an ultrastructural, cryo-EM level, immunogold labelling of GFP clearly depicts GFP-SCIMP labelling on the plasma membrane at sites of protrusions and ruffles (Fig. 1d). Quantification of the gold labelling shows SCIMP to be enriched ∼4 fold in ruffles compared with other stretches of plasma membrane (Fig. 1d). Both the dorsal ruffles and filopodia of macrophages are cholesterol-rich membranes replete with lipid raft microdomains, and these sites are also enriched with immune receptors, including some TLRs^10^,^22^,^23^. Thus SCIMP at these sites is positioned to participate in pathogen detection and/or receptor-mediated activation of macrophages.

**Figure 2:**
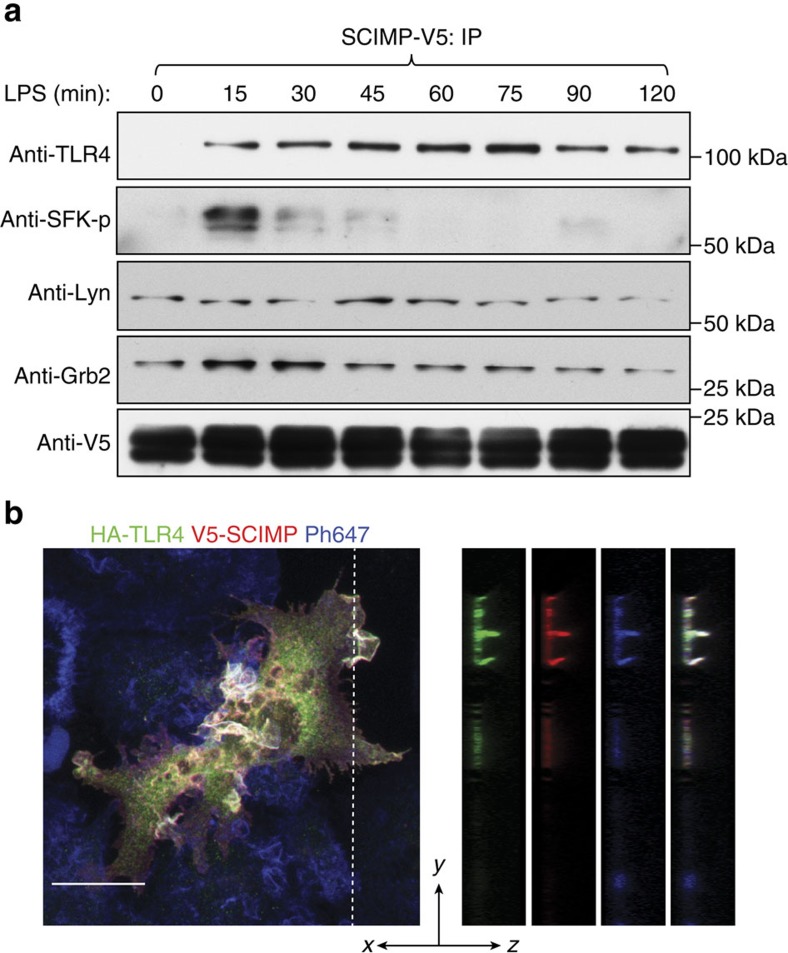
SCIMP is recruited to TLR4 in macrophages on LPS activation. (**a**) V5-labelled SCIMP was stably expressed in RAW264.7 macrophages, cells were treated with LPS, and at the indicated times, the V5 antibody was used for immunoprecipitation. Samples were probed for TLR4, pSFK(Y416), Lyn, Grb2 and V5-SCIMP. (**b**) Confocal imaging of LPS-treated (30 min) RAW264.7 cells transiently co-expressing TLR4-HA (green) and SCIMP-V5 (red), with phalloidin (blue). TLR4 and SCIMP are colocalized (white) on dorsal surface ruffles, as shown for separate channels in the Z projections. Data in **a** and **b** are representative of three independent experiments. The scale bar in **b** represents 10 μm.

To determine whether this adaptor is associated with specific receptor pathways, we performed unbiased screens to identify possible SCIMP binding partners in activated macrophages. GST-SCIMP was used for pull-down assays, along with a protease cleavage and elution strategy^24^, to optimize capture of genuine binding partners from lysates of activated macrophages. LC/MS/MS analysis of SCIMP-bound proteins in LPS-activated macrophages identified the SFK Lyn and the adaptor protein Grb2 (Fig. 1e), which were previously identified as SCIMP partners in B cells^18^. In addition, one of the top hits identified from this analysis was TLR4 (Fig. 1e,f and Supplementary Fig. 1c), the prototypical TLR and a previously unidentified binding partner of SCIMP. To verify this association in cells, we performed immunoprecipitation (with a V5 antibody) from macrophages expressing V5-tagged SCIMP. Consistent with the LC/MS/MS data (Fig. 1f), immunoblotting confirmed the co-immunoprecipitation of Lyn and Grb2, which were constitutively bound to SCIMP (Fig. 2a). In addition, TLR4 co-immunoprecipitated with SCIMP, and in this case, the interaction was strictly LPS-induced (Fig. 2a), occurring rapidly after ligand activation. A phospho-specific antibody recognizing active SFKs (pY416 Src) reveals that LPS acutely and transiently activates an SFK in this complex (Fig. 2a), which is consistent with, and likely to be, Lyn. Thus, we reveal SCIMP as a component of LPS-activated TLR4 complexes in macrophages.

pTRAPs are typically associated with cholesterol-rich membrane microdomains and detergent-resistant fractions. For instance, SCIMP is enriched in tetraspanin-enriched microdomains in B cells^18^. Here we show that in LPS-treated macrophages, SCIMP and Lyn are also enriched in detergent-resistant membrane fractions, as marked by flotillin (Supplementary Fig. 1d). This is consistent with the nature of pTRAPs and with the localization of SCIMP in cholesterol-rich filopodia and ruffles (Fig. 1b–d). This is also significant for the association of SCIMP with TLR4, which is also in lipid raft domains and detergent-resistant fractions^6^. However, the apparent interaction between SCIMP and TLR4 in activated macrophages (Fig. 2a) was not merely a consequence of SCIMP being partitioned into LPS-induced microdomains, since there was no non-specific pull-down of the lipid raft marker flotillin in immunoprecipitates (Supplementary Fig. 1e). Finally, we sought verification that SCIMP and TLR4 colocalize in cells by co-expressing HA-tagged TLR4 and V5-SCIMP in activated macrophages. We have previously shown that TLR4 is concentrated in dorsal ruffles, which act as sites for initiation of signalling in LPS-activated macrophages^23^. Here the joint labelling depicts a concentration of HA-TLR4 in these actin-rich ruffle membranes where it colocalizes with V5-SCIMP (Fig. 2b). Together these results indicate that SCIMP and LPS-activated TLR4 are co-located in plasma membrane domains that are also signalling-competent locales.

### SCIMP exerts selective effects on macrophage TLR responses

**Figure 3:**
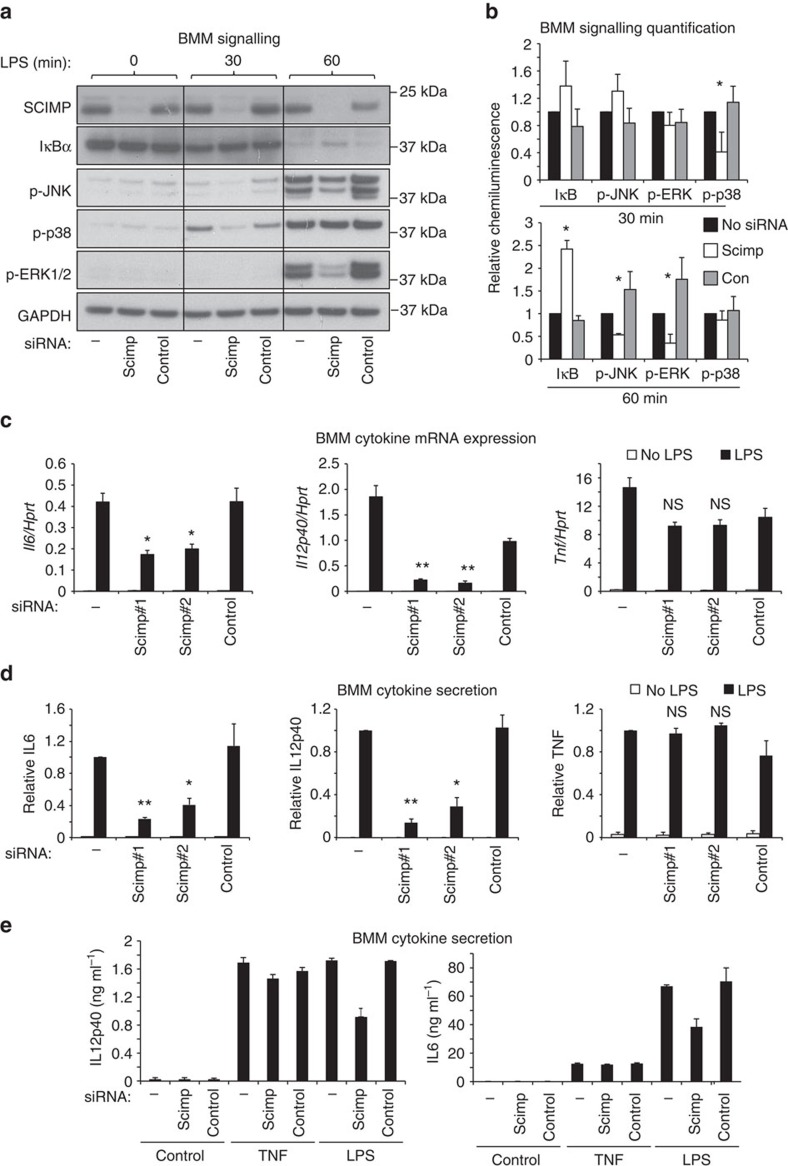
SCIMP is required for a subset of proinflammatory cytokine responses in macrophages. (**a**) SCIMP was silenced by siRNA in BMM. Representative immunoblots showing levels of SCIMP, IκB, phospho-JNK, phospho-p38 and phospho-ERK1/2 at 0, 30 and 60 min post-LPS stimulation. (**b**) Relative chemiluminescence of IκB, phospho-JNK, phospho-ERK1/2 and phospho-p38 was assessed at 30 min and 60 min post-LPS stimulation in SCIMP-silenced BMM. Graphs represent pooled data from *n*=3 experiments (mean+s.e.m.). (**c**,**d**) SCIMP silencing in BMM reduces proinflammatory cytokine production at the mRNA (**c**) and protein (**d**) level. Levels of individual mRNAs, relative to *Hprt*, at 4 h post-LPS stimulation were assessed by qPCR (*n*=4 experiments), and levels of secreted cytokines at 24 h post-LPS stimulation were assessed by enzyme-linked immunosorbent assay (ELISA; *n*=4 experiments). Graphs depict mean+s.e.m. (**e**) IL-6 and IL-12p40 protein levels were assessed by ELISA in SCIMP-silenced BMM treated with LPS or TNF for 24 h. Data is representative of two independent experiments. Graphs depict mean+range from technical repeats (*n*=2). Data in (**a**–**d**) are representative of, or combined from, at least 3 independent experiments. For (**b**–**d**), significance was assessed using one-way ANOVA (*P<0.05 and **P<0.01; NS, not significant).

Given the contributions of other pTRAPs to immune receptor signalling in T and B cells, we next examined a role for SCIMP as a signalling adaptor in its guise as a direct, LPS-induced binding partner of TLR4. The LPS-mediated phosphorylation of ERK, p38 and JNK MAPKs is impaired after small interfering RNA (siRNA) silencing of SCIMP in primary macrophages (BMM). SCIMP-silenced cells also have a modest but significant impairment in LPS-triggered degradation of IκB (Fig. 3a,b). Interestingly, this effect on signalling responses is very transient; for example, SCIMP silencing impairs p38 MAPK activation at 30 min, but not at 60 min, post-LPS, and by 120 min most of the signalling responses are unaffected by SCIMP (Fig. 3a,b and Supplementary Fig. 2a). The transient effect of SCIMP on TLR signalling responses was not explored further, but these findings suggest that SCIMP is likely to have partial or selective effects on downstream biological responses. To test this, we examined LPS-induced cytokine outputs in SCIMP-silenced BMM. Indeed, after depletion of SCIMP with two specific siRNAs, the synthesis and secretion of the proinflammatory cytokines interleukin 6 (IL-6) and IL-12p40 are substantially reduced, while remarkably, tumor necrosis factor (TNF) is unaffected (Fig. 3c,d). These effects are discriminating for TLR signalling, as TNF-inducible IL-6 and IL-12p40 production were unaffected by SCIMP silencing in BMM (Fig. 3e). Further verifying this response, we find that retroviral transduction and overexpression of SCIMP in BMM selectively amplifies LPS-inducible production of IL-12p40, but not of TNF (Supplementary Fig. 2b). Examination of additional cytokines further attests to this selectivity, since levels of the TLR-inducible cytokines IFN-β, IL-10 and IL-12p70 are all unaffected by SCIMP silencing in BMM (Supplementary Fig. 2c). SCIMP additionally does not regulate inducible expression of specific MyD88-dependent (for example, *Il1β* and *Ccl7*) or MyD88-independent (for example, *Ifnβ* and *Cxcl10*) TLR4 target genes (Supplementary Fig. 2d).

Having examined the effects of SCIMP depletion on TLR4-triggered cytokine outputs, we next extended our analysis to examine possible roles downstream of other TLRs. siRNA silencing of SCIMP in BMM reduced the inducible production of IL-6 and IL-12p40, but not TNF, in response to agonists of TLR3 (Poly(I:C)), TLR7 (imiquimod) and TLR1/2 (Pam3CSK4) (Supplementary Fig. 3a–c). As an alternative and complementary approach, we also assessed the effect of CRISPR/Cas9-mediated SCIMP deletion on TLR responses in RAW264.7 cells. As expected, deletion of SCIMP in multiple clonal cell lines (Supplementary Fig. 4a) did not impair either LPS-inducible TNF production (Supplementary Fig. 4b) or LPS-/Pam3CSK4-induced TNF secretion (Supplementary Fig. 4c,d). However, the secretion of IL-6 at the behest of LPS-activated TLR4 or Pam3CSK4-activated TLR1/2 was significantly reduced (Supplementary Fig. 4c,d). The low levels of LPS-inducible IL-12p40 secretion were also reduced, whilst Pam3CSK4-inducible IL-12p40 secretion from RAW264.7 cells could not be detected. Thus, CRISPR/Cas9-mediated SCIMP deletion corroborated the results obtained with siRNA depletion of SCIMP in BMM. Taken together, these findings show that, in the context of an otherwise broad cytokine programme induced by TLRs, the adaptor SCIMP is responsible for driving a uniquely selective subset of key proinflammatory cytokines, namely IL-6 and IL-12p40. To uncover the mechanism for this selective cytokine regulation, we next dissected the interaction between SCIMP and TLR4 in more detail.

### Phosphorylation of SCIMP at Y96 enhances binding to TLR4

**Figure 4:**
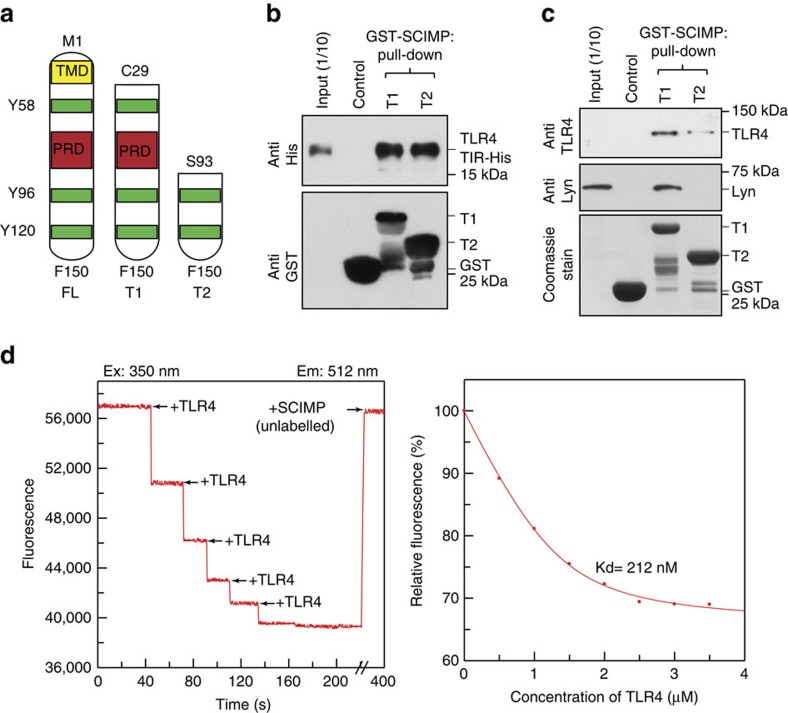
SCIMP interacts directly with TLR4. (**a**) A schematic diagram depicting T1, T2 or full-length (FL) murine SCIMP with transmembrane domain (TMD), PRD, tyrosine residues 58, 96 and 120 (green bars) and N- and C-terminal amino acids. (**b**) Bacterially expressed GST tag alone, or GST-tagged SCIMP T1 or T2 was used to pull-down recombinant His-TLR4-TIR. (**c**) GST-SCIMP T1 or T2 fusion proteins were used in GST pull downs with extracts from LPS-activated RAW264.7 cells and probed for TLR4 and Lyn. (**d**) Titration of fluorescently labelled SCIMP-T1 with TLR4-TIR (left panel), with Kd (212 nM) determined by curve fit analysis (right panel). Data in **b** and **c** are representative of three independent experiments.

The canonical model for adaptor interactions with TLRs is via direct, homotypic interactions between the TIR domains of TLRs and TIR domain-containing adaptor proteins (MAL, MyD88, TRIF, TRAM and SARM)^4^,^5^,^6^. Despite SCIMP lacking a TIR domain, our pull-down experiments suggested at least a close association between SCIMP and TLR4. Like other TRAPs, SCIMP has a short extracellular domain and a long cytoplasmic tail. The intracellular domain contains a proline-rich domain (PRD) and several tyrosine residues that can be phosphorylated by Lyn (refs 11, 18). Two truncated forms of the SCIMP cytoplasmic tail were produced (Fig. 4a), T1 (entire intracellular region: amino acids 29–150) and T2 (C terminal region: amino acids 93–150). The cytoplasmic TIR domain (amino acids 670–835) of TLR4 was also produced, and in *in vitro* pull-downs using recombinant proteins, we find that both GST-SCIMP-T1 and T2 bind to the His-TLR4-TIR domain (Fig. 4b). Thus, the C-terminal TLR4-TIR domain and the C-terminal region of SCIMP (amino acids 93–150) can directly interact. To confirm this binding in cell lysates, GST-SCIMP T1 and T2 were used for pull-downs from LPS-activated macrophage extracts. SCIMP-T1, and to a lesser extent SCIMP-T2, pulled down endogenous TLR4 (Fig. 4c). Importantly, neither GST-SCIMP T1 nor T2 interacted with endogenous TLR4 in extracts of unstimulated macrophages, whereas GST-SCIMP T1 pull-downs show constitutive binding of Lyn (Supplementary Fig. 5a). These data confirm that, whereas SCIMP interacts constitutively with Lyn in macrophages, its interaction with TLR4 is agonist-induced. Moreover, the pull-down experiments (Fig. 4c) confirm binding of TLR4 to the C-terminus of SCIMP, but also suggest that other regions of SCIMP, in addition to S93-F150, contribute to the stronger binding offered by the longer T1 construct under in-cell conditions. Hence, analysis of the interaction between recombinant SCIMP-T1 and recombinant TLR4-TIR (Fig. 4b) is unlikely to capture all of the factors involved in the interactions between these proteins within macrophages. As expected, SCIMP-T2, which lacks the PRD, does not interact with Lyn (Fig. 4c and Supplementary Fig. 5a). Finally, the *in vitro* interaction between SCIMP and TLR4 was analysed in fluorescence binding assays using recombinant, fluorescently labelled SCIMP-T1 and TLR4-TIR (Fig. 4d, left panel); these experiments show that the direct *in vitro* interaction occurs with moderately high affinity (212 nM; Fig. 4d, right panel). Based on these findings, we reveal novel binding, through an unconventional mode, between a non-TIR adaptor and the TIR domain of TLR4.

**Figure 5:**
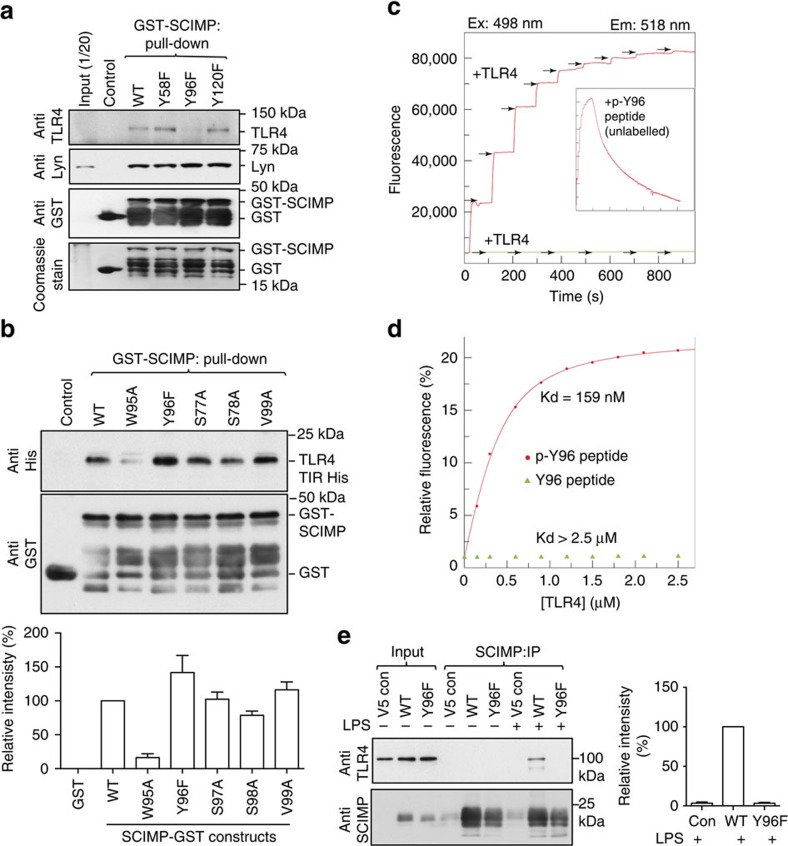
Phosphorylation of SCIMP at Y96 enhances binding to TLR4. (**a**) Pull-down assays using LPS-activated RAW264.7 cell lysates and GST-tagged tyrosine to phenylalanine (Y to F) mutants of SCIMP-T1. SCIMP interactions with either TLR4 or Lyn were assessed by immunoblotting. (**b**) GST pull-down assays were performed using GST-SCIMP mutants and recombinant His-tagged TLR4-TIR domain, and binding was evaluated using immunoblotting against His-TLR4-TIR (upper panel). Quantification of immunoblots was performed using densitometry. Data are combined from two independent experiments (mean+range, lower panel). (**c**) Phosphorylation at Y96 enables SCIMP to bind to TLR4. Titration of fluorescently labelled p-Y96-containing (red) or Y96-containing (green) SCIMP-derived peptides (aa 90–107) with TLR4-TIR. Inset shows fluorescence recovery over a longer time course with unlabelled p-Y96 peptide. (**d**) The Kd (159 nM and>2.5 μM for p-Y96- and Y96-containing peptides, respectively) was determined by curve fit analysis. (**e**) SCIMP phosphorylation at Y96 is required for *in vivo* TLR4 binding. RAW264.7 cells stably expressing wild type (WT) or Y96F SCIMP were used to assess the agonist-induced SCIMP-TLR4 interaction, by co-immunoprecipitation of SCIMP followed by immunoblotting for TLR4 and SCIMP (left). The level of TLR4 pull-down was quantified relative to SCIMP levels (right) to correct for minor differences in levels of WT versus Y96F SCIMP expression (mean+s.e.m.). Data in **a** are representative of three independent experiments, data in **b** are representative of (top) or combined from (bottom) *n*=2 independent experiments, and data in **e** are representative of three independent experiments (left) or combined from *n*=3 independent experiments (right).

As the SCIMP-TLR4 interaction is not only direct (Fig. 4b,d), but also ligand-dependent (Fig. 2a), we next examined whether tyrosine phosphorylation of SCIMP regulates binding. Three tyrosines in the cytoplasmic domain are conserved between human and mouse SCIMP (Fig. 4a). Phosphorylation-deficient mutagenesis of each of these residues reveals that only the Y96F mutation attenuates binding of GST-SCIMP-T1 to endogenous TLR4 in extracts from LPS-activated macrophages (Fig. 5a). By contrast, phosphorylation of these tyrosines is not required for binding to Lyn, which binds to the PRD of SCIMP^18^. Mutation of a residue (W95) adjacent to Y96 in GST-SCIMP-T1 also substantially reduces binding to recombinant His-TLR4-TIR (Fig. 5b), further highlighting the importance of this region of SCIMP for its interaction with TLR4. In contrast, testing the capacity of GST-SCIMP mutants to bind recombinant His-TLR4-TIR (rather than TLR4 from LPS-activated macrophages) shows the (unphosphorylated) Y96F SCIMP mutant still binds TLR4 (Fig. 5b), thus indicating that phosphorylation is not required for *in vitro* binding. These data therefore suggest that extracts from LPS-activated macrophages trigger phosphorylation of GST-SCIMP at Y96, enhancing its binding to endogenous TLR4. Hence, the affinity of the interaction between phosphorylated SCIMP and TLR4 in LPS-activated cells is likely to be much greater than that observed when using recombinant proteins (Fig. 4d). To directly address this, we performed *in vitro* binding assays, using recombinant TLR4-TIR domain and short SCIMP-derived peptides (amino acids 90–107) containing either unphosphorylated or phosphorylated Y96. These experiments show that the SCIMP-derived peptide-containing phosphorylated Y96 binds to TLR4 with much greater affinity than the unphosphorylated peptide (Fig. 5c,d). Finally, the requirement for SCIMP Y96 phosphorylation for *in vivo* binding to TLR4 was tested in cells stably expressing full-length proteins. As previously observed (Fig. 2a), LPS triggers an association between SCIMP and TLR4, however this does not occur with the SCIMP-Y96F mutant (Fig. 5e). Collectively, the above data define SCIMP as a new direct binding partner and non-TIR adaptor for TLR4, and delineate a new recruitment mechanism, in which tyrosine phosphorylation of SCIMP, most likely by Lyn, enables it to directly bind to the TLR4 TIR domain. The consequences of SCIMP binding to TLR4, and the direct involvement of Lyn in these processes, were next examined.

### SCIMP is required for tyrosine phosphorylation of TLR4

The interaction of SCIMP with TLR4 likely underpins SCIMP-mediated regulation of cytokine production, but several possible mechanisms could account for this effect. For instance, LPS induces the endocytosis of surface-activated TLR4, which is critical for eliciting specific cytokine outputs generated by endosomal signalling events that are distinct from those at the cell surface^25^,^26^. To gauge whether SCIMP alters TLR4 endocytosis, the surface expression and ligand-dependent internalization of TLR4 were measured in BMM after siRNA silencing of SCIMP. In these experiments, both basal TLR4 expression and endocytosis of TLR4 were unaffected (Supplementary Fig. 5b,c). We next investigated whether there may be interplay between SCIMP and TIR-containing adaptors. Here we find that, although SCIMP interacts with TLR4 on LPS activation, it does not interact with the proximal TLR4 adaptor MAL, under the same conditions (Supplementary Fig. 5d). Furthermore, SCIMP is still recruited to TLR4 in the absence of MyD88 (Supplementary Fig. 5e). These findings further highlight the distinct nature of the SCIMP-TLR4 interaction, and suggest that other mechanisms likely account for SCIMP-dependent, TLR4-inducible cytokine production.

**Figure 6:**
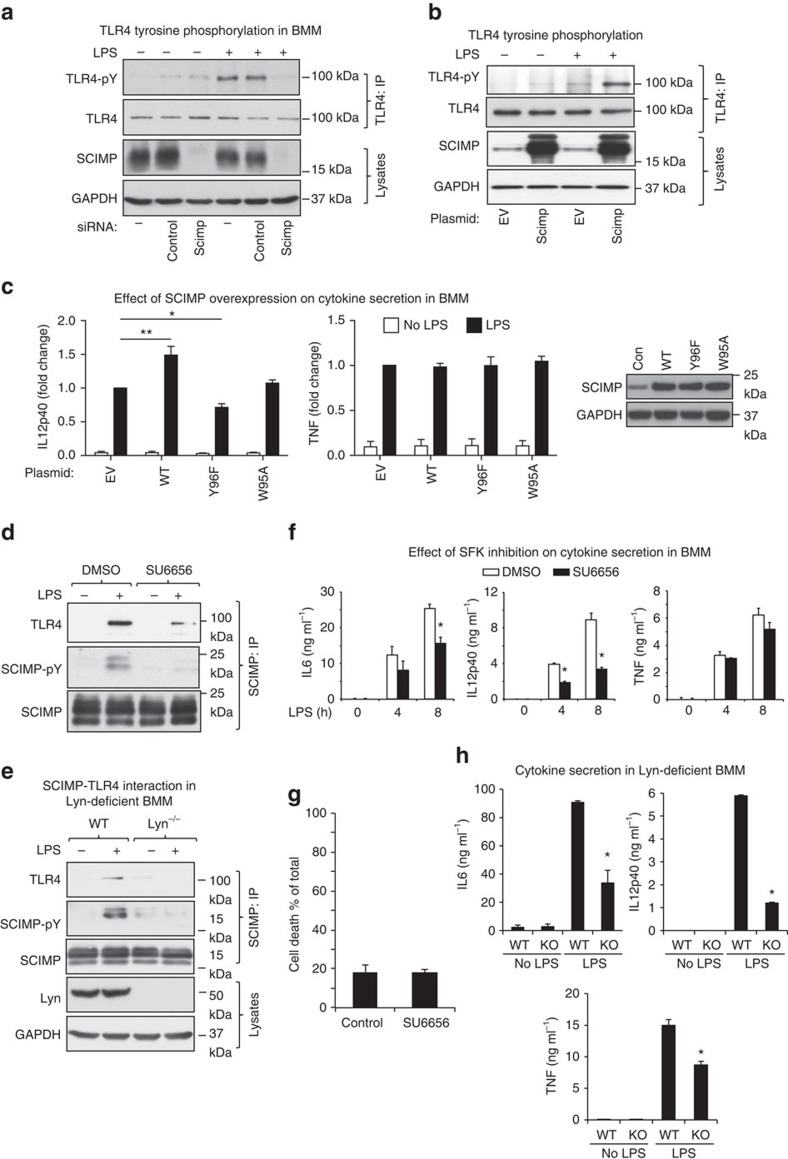
SCIMP drives cytokine selectivity by scaffolding the SFK Lyn for TLR4 phosphorylation. (**a**) siRNA-mediated SCIMP knockdown in BMM reduces tyrosine phosphorylation of TLR4. After SCIMP silencing (lysates, bottom panels), cells were stimulated for 30 min with LPS, TLR4 was immunoprecipitated and blots were probed for anti-phospho-tyrosine. (**b**) SCIMP overexpression increases tyrosine phosphorylation of TLR4. RAW264.7 cells stably transfected with empty vector or SCIMP were treated with 30 min with LPS, after which TLR4 was immunoprecipitated and tyrosine phosphorylation was assessed by immunoblotting. (**c**) IL-12p40 and TNF cytokine production was assessed in primary macrophages (BMM), retrovirally transduced with empty vector (EV), wild-type SCIMP (WT), Y96F SCIMP or W95A SCIMP, at 24 h post-LPS stimulation (mean+s.e.m.). SCIMP protein expression in transduced BMM is shown in the panel on the right. (**d**) The Src kinase inhibitor SU6656 impairs SCIMP tyrosine phosphorylation and its interaction with TLR4, as assessed by SCIMP immunoprecipitation experiments from lysates of RAW264.7 cells stimulated with LPS for 5 min. (**e**) Lyn is essential for LPS-induced SCIMP phosphorylation, as well as the interaction between SCIMP and TLR4 as assessed by SCIMP immunoprecipitation from lysates of WT and *Lyn*^−/−^ BMM stimulated with LPS for 5 min. (**f**) BMM were pre-treated with SU6656 for 30 min and then stimulated with LPS for 4 h or 8 h. Secreted cytokines (IL-12p40, IL-6 and TNF) were measured by enzyme-linked immunosorbent assay (ELISA; mean+s.e.m.). (**g**) The effect of SU6656 on cell death in LPS-activated BMM was assessed using the LDH release assay. LDH in culture supernatants was measured as a percentage of total cellular LDH at 8 h post-LPS stimulation from the same samples as in **f** (mean+s.e.m.). (**h**) Levels of LPS-induced cytokines (IL-6, IL-12p40 and TNF; ELISA) in culture supernatants from wild type (WT) and *Lyn*^−/−^ (KO) BMM were assessed at 24 h post-stimulation (mean+s.e.m.). All experiments were repeated at least 3 times, and all graphical data are pooled from at least *n*=3 independent experiments (mean+s.e.m.). Significance was assessed using one-way ANOVA in **c**, **f** and **h** (*P<0.05 and **P<0.01).

TLR4 (ref. 27), as well as several other TLRs including TLR2 (refs 28, 29, 30), TLR3 (ref. 31) and TLR8 (ref. 32), are tyrosine phosphorylated in a ligand-dependent manner. Given that SCIMP is required for responses to multiple TLRs (Supplementary Figs 3a–c and 4c,d) and since other TRAPs act as adaptors linking SFKs to receptors for phosphorylation^11^, we considered the possibility that SCIMP may be recruited to facilitate the ligand-dependent phosphorylation of TLR4. Indeed, we find that LPS-induced tyrosine phosphorylation of TLR4 is dramatically reduced on siRNA silencing of SCIMP in primary macrophages (Fig. 6a). The opposite effect is apparent when SCIMP is overexpressed in RAW264.7 cells, with markedly increased levels of LPS-induced phospho-TLR4 being observed (Fig. 6b). Thus, the direct interaction of SCIMP with TLR4 facilitates tyrosine phosphorylation of TLR4 in response to LPS, offering a possible mechanism for delineating selective cytokine outputs. To determine whether the SCIMP-TLR4 interaction is indeed required for LPS-inducible, SCIMP-dependent cytokine production, we examined the functional capacity of the SCIMP Y96F and W95A mutants, which do not interact with TLR4 in cells or *in vitro*, respectively (Fig. 5a,b,e). As predicted, these mutants are unable to amplify LPS-inducible production of the SCIMP-dependent cytokine IL-12p40 when overexpressed in primary macrophages (BMM), whereas wild-type SCIMP selectively promotes LPS-inducible IL-12p40 but not TNF production (Fig. 6c). Interestingly, there was a modest but statistically significant decrease in LPS-inducible IL-12p40 production for the Y96F mutant versus empty vector. This could relate to the fact that, although this mutant is unable to be phosphorylated, it can still bind to Lyn (Fig. 5a) and thus may have a dominant-negative effect on this signalling response.

### Lyn phosphorylates SCIMP to enable an interaction with TLR4

In B cells, MHC-II cross-linking triggers SCIMP phosphorylation^18^. In macrophages treated with either LPS or Pam3CSK4, SCIMP was rapidly phosphorylated (Supplementary Fig. 5f), consistent with the requirement for this pTRAP in cytokine responses downstream of multiple TLRs (Supplementary Figs 3a–c and 4c,d). Although SCIMP is constitutively associated with Lyn in macrophages (Fig. 2a), the role of this SFK in TLR signalling is complex, and somewhat controversial. It has been reported to both inhibit and enhance TLR responses in different cell types and in different *in vivo* settings^33^,^34^,^35^,^36^. We therefore more directly assessed the involvement of Lyn in the TLR4-SCIMP pathway. In support of a model in which activated Lyn phosphorylates SCIMP to enable an interaction with TLR4, we found that a SFK inhibitor impairs LPS-induced phosphorylation of endogenous SCIMP, as well as the interaction between SCIMP and TLR4 (Fig. 6d). Moreover, both SCIMP phosphorylation and its inducible association with TLR4 were ablated in BMM from *Lyn*^−/−^ mice (Fig. 6e). Thus, both pharmacological and genetic approaches posit Lyn as an essential upstream kinase for the agonist-induced association between SCIMP and TLRs. A further prediction of this model is that, if SCIMP is unable to bind to TLR4 for its tyrosine phosphorylation, TLR4 signalling for production of SCIMP-dependent cytokines will be impaired. Accordingly, a SFK inhibitor selectively reduces IL-6 and IL-12p40, but not TNF, production from BMM (Fig. 6f), without affecting cell viability (Fig. 6g). Similarly, *Lyn*^−/−^ BMM were compromised for LPS-inducible IL-6 and IL-12p40 production (Fig. 6h). In this case, TNF production was also somewhat reduced, which likely reflects a broader role for Lyn beyond its function in the SCIMP pathway. We thus conclude that Lyn-mediated phosphorylation of immune-restricted SCIMP at Y96 enables an unconventional interaction with the TIR domain of LPS-activated TLR4. SCIMP-mediated phosphorylation of TLR4 then serves as a mechanism to initiate a transient proinflammatory signalling code that selectively produces the inflammatory mediators IL-6 and IL-12p40 in the context of acute inflammatory responses. Given that several TLRs are tyrosine phosphorylated^27^,^28^,^31^,^32^, and that SCIMP directs similar cytokine outputs from multiple TLRs (Supplementary Figs 3a–c and 4c,d), this pTRAP is likely to control other TLR responses through a shared mechanism. Overall, SCIMP is thus revealed as a key, agonist-inducible signalling adaptor and scaffold for phosphorylation of TLRs to enable specific proinflammatory cytokine responses.

## Discussion

In this study, we extend the known range of pTRAP adaptor functions to the control of signalling from the TLR family. The immune-restricted expression of the pTRAP family member SCIMP, particularly in macrophages, and its enrichment in signalling-rich membrane domains, position this adaptor as a controller of TLR-mediated innate immune signalling responses. We identified a unique proinflammatory cytokine signature for SCIMP, which was operational through multiple TLRs. To delineate the nature of SCIMP binding and the mechanism for its role in signalling and cytokine regulation, in-depth studies were carried out on LPS-activated TLR4. The findings reveal that LPS triggers rapid Lyn kinase activation, leading to Lyn-mediated phosphorylation of SCIMP at Y96 and the inducible interaction between TLR4 and SCIMP via unconventional TIR-non-TIR domain binding (see model in Supplementary Fig. 6). The association between TLR4 and SCIMP enables tyrosine phosphorylation of TLR4 (most likely via SCIMP-associated Lyn), thus initiating a transient TLR4 signalling response, particularly in the MAPK signalling arms. This SCIMP-mediated pathway results in the synthesis and secretion of primarily the key proinflammatory cytokines IL-6 and IL-12p40. This fine-tuning of inflammatory signalling pathways represents a remarkable level of selectivity within the broader TLR-dependent cytokine profile elicited by innate immune cells.

Since the mid-1990s, the TLR signalling field has focused almost exclusively on the role of the MyD88, MAL, TRAM and TRIF TIR-containing adaptors in providing signal bifurcation. MyD88, alone or in combination with MAL, drives a MyD88-dependent gene expression programme^37^,^38^. Similarly, TRIF, alone or in combination with TRAM, promotes the so-called MyD88-independent gene expression programme, typified by interferon regulatory factor 3-mediated induction of IFN-β expression^39^,^40^. Recent studies on the TIR-containing BCAP adaptor, which links TLRs to PI3K/Akt activation and negative regulation of cytokine outputs^41^,^42^, have highlighted additional layers of complexity that exist in adaptor-mediated control of TLR responses. Now, our identification of SCIMP as a non-TIR-containing adaptor mediating TLR4 phosphorylation provides a completely new class of TLR adaptor and a new mode of TLR adaptor recruitment for subsequent signalling responses. Importantly, it also defines a new axis for signal bifurcation. SCIMP is an early hierarchical recruit to LPS-bound TLR4, mediating receptor phosphorylation, a transient signalling response and a selective effect on downstream inflammatory outputs. The interplay between SCIMP and TIR-containing adaptors during acute TLR signalling responses is an area of ongoing investigation, however we find that MyD88 is not required for the LPS-inducible association between TLR4 and SCIMP (Supplementary Fig. 5e). This finding is consistent with a recent study, which found no role for MyD88 in SCIMP responses downstream of zymosan^19^. The concentration of SCIMP in cell surface ruffles and projections and its fractionation in detergent-resistant membrane fractions (Fig. 1b,d and Supplementary Fig. 1b,d) are both consistent with the known enrichment of pTRAP family members in lipid raft domains and with the enrichment of TLR4 in membrane ruffles and lipid rafts^6^,^11^,^23^. Moreover, SCIMP is also on intracellular membranes (Supplementary Fig. 1b), and its activation (Supplementary Fig. 5f) and requirement for cytokine production downstream of multiple TLRs (Supplementary Figs 3a–c and 4c,d) suggests that it is likely to function from both endosomal environments (for example, TLR3 and TLR7) and from the cell surface (for example, TLR1/2).

Despite the central role of tyrosine phosphorylation of receptors in signal transduction pathways, only a limited number of studies have investigated this in the context of TLRs. TLR2 (refs 29, 30), TLR3 (ref. 43) and TLR4 (refs 27, 44, 45) are all known to be tyrosine phosphorylated in an agonist-induced manner. In the case of TLR4, Lyn kinase has been chiefly implicated as the responsible kinase^27^, although Syk is also reported to phosphorylate TLRs (ref. 46). Our findings here identify SCIMP as an essential mediator of LPS-induced TLR4 tyrosine phosphorylation (Fig. 6a,b), and Lyn as being a positive regulator of TLR4 signalling (Fig. 6f,h) that is indispensible for the SCIMP-TLR4 interaction (Fig. 6d,e). Our data thus supports a model in which Lyn is required for TLR4 tyrosine phosphorylation in macrophages, although it is likely that other tyrosine kinases may contribute to and diversify patterns of TLR4 phosphorylation, for example, to provide cell-type-selectivity to such responses. Our discovery of the SCIMP-Lyn-TLR4 axis may also help explain some of the apparently contrasting roles for Lyn in TLR signalling responses. For example, Lyn has been reported to inhibit TLR-mediated inflammatory responses in macrophages^34^, but promote similar responses in dendritic cells^35^,^36^. Under our experimental conditions, Lyn promotes LPS-inducible IL-12p40 and IL-6 production in macrophages (Fig. 6f,h), which is also consistent with our functional data on SCIMP (Fig. 3c–e). Given that SCIMP constitutively associates with Lyn in macrophages (Fig. 2a), its presence or absence in a particular cellular context is likely to be a critical determinant of how Lyn participates in TLR responses.

Despite knowledge of specific tyrosine kinases that mediate TLR phosphorylation, how this process is actually initiated was previously unknown. Our studies on SCIMP have solved this puzzle by unravelling this pTRAP member as an essential adaptor directly linking Lyn to TLR4 in macrophages. Previous studies have shown that Y674 within human TLR4 (mouse equivalent: Y672) is necessary for TLR4 dimerization and TIR-mediated adaptor recruitment^27^. Such effects are predicted to have profound and more widespread effects on subsequent signalling responses, so it seems unlikely that SCIMP exerts its effects by promoting phosphorylation of this particular residue. Thus, TLR4 phosphorylation at Y674 may represent part of the tonic signal, required for more widespread licensing of TLR4 signalling responses in macrophages. In contrast, a previous study by Ronni *et al*.^47^ identified surface-exposed residues within the TLR4 TIR domain that can contribute to selective transcriptional regulation, including for the *IL-12p40* promoter. Recruitment of specific signalling complexes to phosphorylated TLR4 is thus predicted to mediate the specific SCIMP-dependent transcriptional programme, although this remains to be formally demonstrated. Given the effect of SCIMP in driving a transient MAPK signalling response (Fig. 3a,b and Supplementary Fig. 2a), it is notable that SCIMP is also associated with the Grb2 adaptor protein (Figs 1e,f and 2a) that is linked to MAPK signalling responses^18^. This therefore represents one candidate signalling component of the SCIMP-dependent TLR4 response. Others include SLP adaptors, to which SCIMP is also known to bind^18^. Importantly, a functional connection between SCIMP-initiated MAPK signalling and downstream biological responses on TLR engagement is yet to be demonstrated. More in-depth studies of SCIMP-dependent TLR signalling are thus required to understand the precise molecular mechanisms by which this adaptor imparts gene-specific control of TLR responses. Nonetheless, our discovery that SCIMP functions downstream of several TLRs (Supplementary Figs 3a–c and 4c,d) broadens its likely biological significance to inflammatory responses, and puts forth SCIMP-mediated tyrosine phosphorylation as a shared mechanism across multiple TLRs.

Through protein–protein interaction studies, we demonstrate that TLR4 and SCIMP uniquely and directly interact (Fig. 4b), and, in cells, this interaction is agonist-induced (Figs 2a, 5e and 6d,e). Mutational analysis delineated W95 and Y96 as critical residues required for the interaction (Fig. 5), the latter in a phosphorylation-dependent manner. Using recombinant proteins, in which Y96 was not phosphorylated, we demonstrated that SCIMP bound to TLR4 with moderately high affinity (Fig. 4d). However, this interaction between the recombinant proteins clearly cannot capture all of the factors involved in cells, where their proximity within membrane sub-domains and post-translational modifications will also be important. Indeed, on the basis of binding studies with peptides (Fig. 5c,d), we predict that the binding affinity between SCIMP and TLR4 would be greatly enhanced on SCIMP phosphorylation at Y96, as occurs upon LPS activation. Interestingly, the C-terminal T2 SCIMP protein (amino acids 93–150) showed a reduced capacity to pull-down TLR4 from activated macrophage cell extracts, by comparison to a protein encompassing the entire intracellular domain (T1, amino acids 29–150; Fig. 4c). This suggests that other residues distal to the region around W95 and Y96 are likely involved in binding to the TLR4 TIR domain. Hence, multiple regions in the cytosolic domain of SCIMP must be involved in supporting in its binding to TLR4.

Innate immune cells produce a wide range of inflammatory cytokines. SCIMP appears to drive the TLR4-dependent production of primarily two of these, namely IL-6 and IL-12p40. Why only these cytokines are independently controlled is now an open question. However, it is notable that both IL-6 and IL-12p40 control T helper (Th) cell differentiation programs. IL-6 promotes Th17 cell differentiation and limits T regulatory cell development^48^, IL-12 directs Th1 cell differentiation, and IL-12p40 homodimers can repress Th1 development^49^. As SCIMP drives production of IL-6 and IL-12p40 (Fig. 3c,d), but not IL-12p70 (Supplementary Fig. 2c), this may provide a molecular switch favouring Th17 over Th1 differentiation through the generation of IL-12p40 homodimers and IL-6. The first published study on SCIMP identified a role in MHC class II signalling^18^. Thus, an attractive model is that SCIMP may serve dual adaptor roles in coordinating Th development programs; first by promoting TLR-inducible expression of cytokines that regulate Th development, and second by modulating MHC class II signalling during antigen presentation^18^. In such a scenario, SCIMP would be predicted to be a key player in shaping adaptive immune pathways, particularly given that it controls the production of Th polarizing cytokines downstream of multiple TLRs (Supplementary Fig. 3a–c).

The identification of a TRAP that relays TLR responses now brings this family of innate immune receptors in line with T and B cell receptors, which are well known for partnering with other TRAP family members to control signalling outputs in lymphocytes. Through the identification of a very distinct non-TIR adaptor pathway, our study highlights how the SCIMP signalling complex mediates TLR4 tyrosine phosphorylation to relay and customize downstream signalling. In so doing, we have delineated proximal innate immune cell-specific signalling events that initiate early signal bifurcation from TLR4, as part of a more comprehensive signalling mechanism that is yet to be fully elucidated (for example, via Grb2 and SLP adaptors), to drive selective transcriptional responses and cytokine outputs. This molecular mechanism involving immune-restricted SCIMP can thus contribute to the exquisite specificity and potency of innate immune cells in generating specific inflammatory cytokine outputs for sculpting of appropriate inflammatory responses. The accompanying predictions that SCIMP will be important as a disease regulator are in keeping with emerging links between *SCIMP* and inflammation-related diseases^20^,^21^.

## Methods

### Antibodies and reagents

The SCIMP antibody was produced by the Walter and Eliza Hall Institute antibody facility. GST-SCIMP-T1 was expressed in *Escherichia coli* (*E. coli*). After PreScission protease cleavage of the GST tag, the SCIMP intracellular domain (amino acids 29–150) was further purified by gel filtration using a Superdex S75 column (GE healthcare) and was used for immunizing rabbits and antibody production. Primary antibodies recognizing His-tag (2366, clone number 27E8), Grb2 (3972, lot 5), p56 Lyn (2796, clone number C13F9), phospho-Src Family (Tyr416; 6943, clone number D49G4), phospho-p38 MAPK (Thr180/Tyr182; 4511, clone number D3F9), IκBα (9242, lot 6), phospho-SAPK/JNK (Thr183/Tyr185; 9251, lot 8) and phospho-ERK1/2 (4370, clone number D13.14.4E) were purchased from Cell Signaling Technology (Beverly, MA, USA). The GST antibody (71–7,500, clone number 1610744A) was purchased from Invitrogen Australia. The anti-flotillin antibody (610820, clone number clone 18) was purchased from BD BioSciences. The anti-actin antibody targeting the N-terminal two thirds of the protein (MAB1501, clone number C4) was purchased from Millipore Australia. The anti-TLR4 antibody targeting TLR4 amino acids 100–200 (ab22048, clone number 76B357.1), used for immunoprecipitation and western blotting, was from Abcam. For cell surface TLR4 staining by flow cytometry, an APC-conjugated anti-mouse CD284 (TLR4) antibody (145405, clone number SA15–21) from BioLegend was used (0.5 μg per million cells). The mouse anti-glyceraldehyde-3-phosphate dehydrogenase antibody (2275-PC-1) was purchased from Trevigen (Gaithersburg, MD, USA). The mouse anti-HA antibody was purchased from Covance (MMS-101P, clone number 16B12), and the mouse anti-V5 antibody (MCA1360, clone number SV5-PK1) was purchased from Abacus ALS. The GFP antibody (A6455, lot 71BH) was purchased from Life Technologies Australia (Scoresby, Victoria, Australia). All primary antibodies were used at a 1:1,000 dilution for immunoblotting (with the exception of those against phospho-p38 (1:500), IκBα (1:500) and phospho-ERK1/2 (1:650)), at a 1:100 dilution for immunofluorescence staining (with the exception of the antibody against V5 (1:500)), at a 1:200 dilution for immunogold staining, and at a 1:50 dilution for immunoprecipitation, unless stated otherwise. Donkey anti-mouse Alexa Fluor 594- (A-21203), donkey anti-rabbit Alexa Fluor 488- (A-21206) and 647- (A31573) conjugated secondary antibodies, Rhodamine Phalloidin (R415) and Alexa Fluor 647 Phalloidin (A22287) were purchased from Molecular Probes (Thermo-Fisher, CA, USA). 4′,6-Diamidino-2′-phenylindole dihydrochloride was purchased from Sigma-Aldrich. Horseradish peroxidase-conjugated goat anti-mouse and rabbit antibodies (81–6520) were obtained from Zymed (San Francisco, CA, USA). All secondary antibodies were used at a 1:10,000 dilution, unless stated otherwise. Sequencing grade, modified trypsin (V5111) was purchased from Promega Australia. SU6656 (572635) was purchased from Merck Australia and was used at a final concentration of 5 μM. Bacterial LPS, purified from *Salmonella enterica* serotype Minnesota Re 595, was purchased from Sigma-Aldrich. The TLR agonists Poly(I:C) (Integrated Sciences), Imiquimod (Integrated Sciences) and Pam3CSK4 (Life Research) were used at 30 μg ml^−1^, 20 μg ml^−1^ and 15 ng ml^−1^, respectively. All other chemicals and reagents were from Sigma-Aldrich, unless otherwise stated.

### DNA constructs and protein expression

The mouse SCIMP full-length construct was amplified by PCR from cDNA (with and without stop codon) and cloned into the pEF6/V5-His TOPO TA and pEGFP-C1 expression vectors. Y96F and W95A mutants of full-length SCIMP were generated by PCR site-directed mutagenesis and were confirmed by sequencing. The wild type and mutant SCIMP sequences were then subcloned into the pMIG retroviral vector. GST-SCIMP T1 (amino acid 29–150) and T2 (amino acid 93–150) were subcloned into pGEX6p-1 (GE Healthcare Life Sciences). SCIMP-T1 (Y58F, W95A, Y96F, S97A, S98A, V99A and Y120F) were generated using the QuikChange site-directed mutagenesis kit (Stratagene) and confirmed by DNA sequencing. Sanger sequencing was performed through the Australian Genome Research Facility (AGRF, Brisbane, Australia). Mouse TLR4-TIR (amino acid 670–835), codon-optimized for bacterial expression, was purchased from Genscript USA. It was then subcloned in to the pET28a vector. All GST fusion proteins were expressed in *E. coli* and purified using glutathione-Sepharose beads (Amersham Biosciences), and all 6 × His fusion proteins were expressed in *E. coli* and purified using Ni-NTA-super flow beads (QIAGEN), according to the manufacturer's instructions. The TLR4-HA construct was kindly provided by Prof. Bostjan Kobe (School of Chemistry and Molecular Biosciences, University of Queensland). MAL was subcloned into pEGFP-N1 from MAL-Cerulean, a gift from Nicholas J. Gay, University of Cambridge, UK.

### Cell culture and transfection

A University of Queensland institutional animal ethics committee approved all animal experimentation. Bone marrow cells were collected from femurs and tibias of 6–8-week-old specific pathogen-free C57Bl/6 mice. *Lyn*^−/−^ mice (on a C57Bl/6 background), used in these studies, have previously been described^50^. In brief, BMM were obtained by *in vitro* differentiation of mouse bone marrow cells in RPMI 1640 supplemented with 2 mM L-glutamine (GlutaMAX), 10% heat-inactivated foetal bovine serum, 50 U ml^−1^ penicillin and 50 μg ml^−1^ streptomycin (Thermo-Fisher) with recombinant CSF-1 (10,000 U ml^−1^, a gift from Chiron), as described previously^51^,^52^. The muscle was removed from the femoral and tibial bones, and bones were cleaned with 70% ethanol. The bone cavity was flushed with complete media and a 23G needle. Pooled bone marrow cells were cultured on bacteriological Sterilin plates (ThermoFisher, Newport, UK; ∼1–2 plates per bone), and differentiated BMM were used on day 6 or 7. Differentiating BMM were replenished with fresh CSF-1 and medium on day 5. Cell culture conditions for all cell lines used in this study, including RAW264.7, NIH3T3, L929, EL4, MOPC and WR19M cells, have previously been described^53^. Immortalized MyD88-deficient BMM (generously provided by Dr Ashley Mansell, Hudson Institute of Medical Research, Melbourne, Australia) were cultured in the same media as BMM, except without the addition of CSF-1. RAW264.7 empty vector and SCIMP stable cell lines (wild type and Y96F mutants) were generated by electroporation. 5 × 10^6^ cells were transfected with 10 μg of plasmid DNA at 240 V, 1,000 μF and ∞ Ω. 48 h after transfection cells were selected using blasticidin (Thermo-Fisher). Transient transfection of RAW264.7 macrophages was performed using lipfectamine 2000 (Thermo-Fisher), according to the manufacturer's instructions.

### Immunoblotting

For immunoblot analysis, cells were lysed in lysis buffer (20 mM Tris, pH 7.4, 100 mM NaCl, 1% NP-40 (Sigma)) with addition of cOmplete protease inhibitors (Roche Applied Science) and phosSTOP tablets (Roche Applied Science). Lysates were transferred to a polyvinylidene difluoride membrane (BioTrace, NZ, USA), blocked with 5% skim milk in phosphate-buffered saline (PBS)/0.1% Tween-20 buffer (TBS-T) and incubated overnight at 4 °C with primary antibodies. After washing, membranes were incubated with a secondary antibody for 1 h, then developed with a chemiluminescence reagent (ECL, Detection Reagents, Thermo Scientific), according to the manufacturer's instructions. Blots were quantified either by Chemiluminescence using the ChemicDoc and Image Lab Software (Bio-Rad Laboratories, Hercules, CA, USA) or by densitometry of X-ray film (FUJIFILM, Tokyo, Japan) using ImageJ version 1.43 (National Institutes of Health, MD, USA). Uncropped images of all immunoblots are presented in Supplementary Figs 7–9.

### Cell surface TLR4 staining and flow cytometry

BMM were harvested in PBS containing 5 mM EDTA and 1% foetal bovine serum and blocked for 20 min on ice with 2.4G2 (anti-CD16/CD32 antibody; Thermo Fisher). Cells were then stained for 20 min on ice with an anti-TLR4 antibody directly conjugated to APC, washed twice in ice-cold PBS containing 5 mM EDTA and 1% foetal bovine serum, resuspended in 100 μl of the same wash solution, and then analysed by flow cytometry using a FACS Canto II (BD) to assess cell surface TLR4 levels.

### siRNA knockdown

Day 6 or 7 BMM were harvested and washed twice in media. Cells were resuspended in complete media at a concentration of 5 × 10^6^ cells/350 μl, and 10 μl 1 M HEPES (tissue culture grade) per ml of cells was added. 350 μl of the cell suspension was transferred to 0.4 cm electroporation cuvettes and mixed with siRNAs against SCIMP or HDAC1 (control gene) to a final concentration of 0.5 μM or tissue culture grade water (no siRNA control) in a final volume of 400 μl. Cells were electroporated at 240 V, 1,000 μF and ∞ Ω. After electroporation, cells were washed twice, counted and then plated at the appropriate cell number. Cells were treated with indicated stimuli at either 24 or 48 h post-transfection. Sequences of siRNAs used are: mScimp #1: sense sequence: 5′-AGACAACCCUCAGCUUGGUACUCAU-3′; antisense sequence: 5′-AUGAGUACCAAGCUGAGGGUUGUCU-3′; mScimp #2: sense sequence: 5′-CAACCACCGAAACCCAGCACUCUAA-3′; antisense sequence: 5′-UUAGAGUGCUGGGUUUCGGUGGUUG-3′; control (mHdac1 #1): sense sequence: 5′-GAACUACCCACUGCGAGACGGCAUU-3′; antisense sequence: 5′-AAUGCCGUCUCGCAGUGGGUAGUUC-3′.

### CRISPR/Cas9-mediated gene knockout

CRISPRs were designed to target the second exon of SCIMP, 77 nucleotides downstream of the first ATG, using the Zhang Lab (MIT) CRISPR design tool ( http://crispr.mit.edu). S.p. Cas9 Nuclease 3NLS (Cat No. 1074181), Alt-R CRISPR crRNA targeting SCIMP (5′-GCCACCTGCAGACACAGTAC-3′) and Alt-R CRISPR tracrRNA (Cat No. 1072532) were purchased from Integrated DNA Technologies, Singapore. RAW264.7 cells were transfected with Cas9, hybridized crRNA and tracrRNA, along with a vector encoding neomycin resistance for transient selection. Transfections were performed using CRISPRMAX transfection reagent from Thermo-Fisher (Cat No. CMAX00003). After G418 selection for 3 days, colonies were expanded and tested by western blot for SCIMP knockout. Matched control lines were generated by omitting the SCIMP specific crRNA from the transfection procedure.

### Gene overexpression by retroviral transduction

Retroviruses were generated in the PlatE virus packaging cell line (ATCC). PlatE cells were maintained in DMEM with 2 mM L-glutamine (GlutaMAX), 10% heat-inactivated foetal bovine serum and 50 U ml^−1^ penicillin and 50 μg ml^−1^ streptomycin. PlatE cells were transfected with retroviral expression constructs encoding wild-type SCIMP or specific SCIMP mutants (Y96F, W95A) using lipofectamine 2000 (Thermo Fisher). Media was changed at 24 h post-transfection, after which cells were cultured at 32 °C for 48 h. At 72 h post-transfection, supernatants containing viral particles were collected and filtered through a Millex-HV PVDF syringe filter. Day 2 murine bone marrow cells were subjected to spin infection at 1,000 g for 2 h at 32 °C. At 48 h post spin infection, media was replaced, and after another 48 h, cells were harvested and used for experiments.

### Quantification of mRNA expression

RNA was harvested from cells using the Direct-zol RNA MiniPrep with TRI-reagent (Zymo Research, Irvine, CA, USA). RNA concentrations were determined by nanodrop ND1000 (Thermo scientific). one microgram of the resulting RNA was DNaseI-treated using amplification grade DNaseI (Life technologies), and then converted to cDNA using oligo dT primers and Superscript III (Life technologies). Levels of mRNA for specific genes were quantified by SYBR green qPCR using the Applied Biosystems ViiA7 Real Time PCR system (Life technologies); 15 s at 95 °C and 1 min at 60 °C for 45 cycles. mRNA levels of individual genes, relative to hypoxanthine guanine phosphoribosyltransferase (*Hprt*), were determined using the ΔCt method. All primers used for quantitative PCR are listed in Supplementary Table 1.

### Protein–protein interaction studies and mass spectrometry

GST-SCIMP sepharose beads were incubated with cell lysates from control or 30 min LPS-activated RAW264.7 macrophages for 1 h at 4 °C with agitation. MicroSpin columns (#27- 3565-01; GE Healthcare) were used for all pull-downs. Beads were washed with ice-cold wash buffer (20 mM Tris, 150 mM NaCl, 1% NP-40, pH 7.4). Elution was achieved conventionally by boiling in 2 × sodium dodecyl sulfate polyacrylamide gel electrophoresis (SDS–PAGE) sample buffer for 5 min. Macrophage extracts were prepared by lysis in ice-cold lysis buffer (20 mM Tris, pH 7.4, 150 mM NaCl, 1% NP-40 (Sigma), cOmplete protease inhibitors (Roche Applied Science), and PhosSTOP tablets (Roche Applied Science). The lysates were centrifuged at 75,600 *g* for 15 min at 4 °C. Supernatants were pre-cleared by the addition of GST-Sepharose beads for 1 h, pelleted at 50 g for 5 min at 4 °C, after which supernatants were collected. GST-tagged recombinant proteins were then incubated with an equal amount of cell lysate at 4 °C for 1 h. Beads were washed extensively with ice-cold 20 mM Tris pH 7.4 containing 150 mM NaCl, 1 mM DTT, 1% NP-40 and 1 mM PMSF, eluted in 2 × SDS–PAGE sample buffer, resolved on 10% SDS–PAGE gels, and stained with Coomassie blue G250. Samples were analysed by either immunoblotting or liquid chromatography–mass spectrometry (LC MS)/MS.

LC MS/MS analysis was performed on a Shimadzu Prominence Nano HPLC (Japan) coupled to a Triple TOF 5600 mass spectrometer (ABSCIEX, Canada) equipped with a nano electrospray ion source (IMB Mass Spectrometry Facility, The University of Queensland). Sample preparation and analysis was performed as previously described^24^. Extracts (6 μl) were injected onto a 50 mm × 300 μm C18 trap column (Agilent Technologies, Australia) at 30 μl min^−1^. The samples were desalted on the trap column for 5 min using 0.1% formic acid (aq) at 30 μl min^−1^. The trap column was then placed in-line with the analytical nano high-performance liquid chromatography column and a 150 mm × 75 μm 300SBC18 column (Agilent Technologies, Australia) for mass spectrometry analysis. Linear gradients of 1–40% solvent B over 35 min at 300 nl min^−1^ flow rate, followed by a steeper gradient from 40 to 80% solvent B in 5 min were used for peptide elution. Solvent B was held at 80% for 5 min for washing the column and returned to 1% solvent B for equilibration, before injection of the next sample. Solvent A consisted of 0.1% formic acid (aq) and solvent B contained 90/10 acetonitrile/0.1% formic acid (aq). The ion spray voltage was set to 2,400 V, declustering potential (DP) 100 V, curtain gas flow 25, nebuliser gas 1 (GS1) 12 and interface heater at 150 °C. The mass spectrometer acquired 500 ms full-scan time-of-flight mass spectrometry (TOF-MS) data followed by 20 by 50 ms full-scan product ion data in an information-dependent acquisition mode. Full-scan TOF-MS data were acquired over the mass range 350–1,400 and for product ion ms/ms 80–1,400. Ions observed in the TOF-MS scan exceeding a threshold of 100 counts and a charge state of +2 to +5 were set to trigger the acquisition of product ion, ms/ms spectra of the resultant 20 most intense ions. The data were acquired and processed using Analyst TF 1.6.1 software (ABSCIEX, Canada). Proteins were identified by database searching using ProteinPilot v4.5 (ABSCIEX, Canada) against the UniProt_Sprot_20130205 database (∼106,000 entries of all species searched, FDR of 1%). Search parameters were defined as a thorough search using trypsin digestion, iodoacetamide cysteine alkylation and all entries in the database. The mass spectrometry score is defined as a measurement of peptide confidence from the ProteinPilot. Scoring algorithm: score=−log(1-(PercentConfidence/100)). For example, a score of 2=99% confidence. %Cov (coverage) refers to the percentage of all identified peptide(s) relative to total amino acid sequence, whereas %Cov (50%) and %Cov (95%) refer to peptide coverage with 50 and 95% confidence, respectively. Proteins were considered as an identified hit if there was at least one peptide identified with 99% confidence.

Co-immunoprecipitation experiments were performed as described previously^23^. Briefly, cells were lysed by passage through successively smaller needles in lysis buffer (20 mM Tris pH 7.4, 140 mM NaCl, 1% NP-40, 1 mM PMSF, 1 mM DTT, Complete Protease inhibitors (Roche Applied Science) and phospho-Stop tablets (Roche Applied Science)). The supernatant was then collected after centrifugation at 14,000 *g* for 15 min and used as the input. For immunoprecipitation, cell lysates were incubated with GFP Nanotrap or appropriate antibodies and protein G agarose beads (Thermo Fisher Scientific) for 1 h at 4 °C. Beads were then washed with excess lysis buffer and bound proteins were solubilized in SDS–PAGE sample buffer. Proteins were separated by 10% SDS–PAGE and analysed by immunoblotting.

### Kinetic analyses of the TLR4-SCIMP interaction

The interaction of SCIMP with TLR4 was monitored using a Fluoromax-4 spectrofluorometer (HORIBA Scientific), and was performed as previously described^54^. Briefly, SCIMP-T1 was labelled with BODIPY FL Iodoacetamide (ThermoFisher Scientific), according to the manufacturer's instructions. Labelled SCIMP was excited at 350 nm, and detection was through a cutoff filter at 512 nm. Fluorescently labelled SCIMP-T1 was titrated with unlabelled TLR4-TIR for the kinetic analysis. The FAM-labelled phosphorylated and unphosphorylated SCIMP Y96 peptides [^90^GQPSAW(p)YSSVKKVRNKKV^107^, or ^90^GQPSAWYSSVKKVRNKKV^107^] were synthesized by Genescipt USA. The excitation and emission wavelengths used were 498 mm and 518 nm, respectively. Fluorescently labelled SCIMP Y96 was titrated with unlabelled TLR4-TIR for the kinetic analysis. The data obtained were fitted using the programme Grafit. All fluorescence measurements were performed at 25 °C in 30 mM Tris, pH 7.4, 150 mM NaCl and 1 mM dithiothreitol.

### Sucrose density gradient separation

Sucrose density gradient separation of membrane extracts was performed as previously described^55^. Briefly, RAW264.7 cells were lysed in homogenization buffer (1% Triton X-100, 10 mM Tris, pH 7.5, 150 mM NaCl, 5 mM EDTA), and the lysate was centrifuged at 2,000 *g* for 2 min at 4 °C to pellet unbroken cells and nuclei. Supernatants were loaded onto a 45 to 5% discontinuous sucrose gradient and centrifuged at 200,000 *g* in a TLS-55 rotor at 4 °C for 18 h. Each fraction was collected from the top of the gradient and subjected to SDS–PAGE separation, and was then analysed by immunoblotting.

### ELISA and Legendplex cytometric bead arrays

Enzyme-linked immunosorbent assays were performed to quantify levels of IL-6, IL-12p40 and TNF secreted from BMM. 96-well maxisorp plates (Thermo Scientific) were coated with 50 μl primary antibody in 0.1 M sodium bicarbonate at pH 9.6 overnight at 4 °C. Plates were then washed with phosphate buffered saline (PBS)/0.05% Tween-20 (PBST), blocked with 200 μl blocking buffer (10% FCS in PBS) for 2 h at 37 °C and then incubated with 100 μl standards or samples overnight at 4 °C. Plates were again washed with PBST, treated with 50 μl secondary antibody made up in blocking buffer for 1 h at 37 °C, washed with PBST, 100 μl extravidin-peroxidase (1:1,000) was added, then samples were incubated for 20–30 min at 37 °C. Peroxidase activity was measured colorimetrically by adding 50 μl 3,3′5,5′-Tetramethylbenzidine substrate (Sigma-Aldrich) and the reaction was stopped by the addition of 50 μl of 2 M H_2_SO_4_. The absorbance was read at 450 nm using a Powerwave XS plate reader and the sample concentrations were calculated by extrapolation from a quadratic curve analysis of the standards. LEGENDplex bead-based immune assays (BioLegend) were performed to determine secreted levels of IFNβ, IL-10 and IL-12p70 in culture supernatants. Experiments were performed as per the manufacturer's protocol, and data were captured using a FACS Canto II flow cytometer (BD). Analysis of the data were performed using the LEGENDplex software with the default settings.

### Fluorescence imaging and electron microscopy

Cells fixed in 4% paraformaldehyde (PFA) and solubilized with 0.1% Triton X-100 for 5 min were immunolabelled with the SCIMP antibody, followed by an Alexa488-conjugated anti-rabbit IgG. Wide-field imaging of endogenous SCIMP was taken with a 12-Mp differential contrast camera (DP71; Olympus) on an upright microscope (BX-51; Olympus) fitted for a 60 × NA 1.35 oil objective using the associated DPController software (version 2.1; Olympus). Confocal imaging of V5-tagged SCIMP in transfected cells was performed using the Ziess LSM 710 confocal microscope fitted with a LD C-Apochromat × 63/1.15 W objective. For cryo-immuno-electron microscopy and gold labelling, GFP-SCIMP-transfected RAW264.7 cells were fixed in 4% paraformaldehyde, embedded in warm gelatin and infused with polyvinylpyrrolidone (PVP)/sucrose overnight and then frozen in liquid nitrogen. Ultrathin cryosections were collected onto formvar-coated grids and immunolabelled with a GFP antibody using standard protocols followed by different sized Protein A-gold secondary antibodies (kindly provided by J. Slot, University of Utrecht, Utrecht, the Netherlands). Sections were viewed on a JEOL 1011 electron microscope (JEOL Australasia, Brookswater, Australia) at 80 kV, and images were captured using the iTEM analysis programme (Soft Imaging System, Olympus, Berlin, Germany).

### Cytotoxicity assays

The effect of SU6656 on BMM viability was assessed using the CytoTox 96 Non-Radioactive Cytotoxicity Assay (Promega). Cell culture supernatants were collected at 8 h post-LPS treatment, centrifuged at 500 *g* for 5 min and lactate dehydrogenase (LDH) release was assessed. Total LDH was assessed by lysing control cells with 0.1% Triton X-100. The percentage of macrophage cell death was then calculated as [(LDH_treatment_—background)/ (LDH_Total_—background)] × 100.

### Statistics

If not stated otherwise, data are presented as arithmetic means±s.e.m. For direct comparison of one experimental variable, a Student's *t*-test was used. For data sets containing multiple comparisons, a one-way analysis of variance (ANOVA) was performed, with Dunett's post-test. All data sets subjected to statistical analysis were compiled from 3 or more independent experiments. In all statistical analyses, a *P* value<0.05 was considered as statistically significant. Statistics were calculated using GraphPad Prism version 7.0 (GraphPad Software, San Diego, CA, USA).

### Data availability

The authors declare that the data supporting the findings of this study are available within the article and its Supplementary Information Files, or are available from the corresponding authors upon request.

## Additional information

**How to cite this article:** Luo, L. *et al*. SCIMP is a transmembrane non-TIR TLR adaptor that promotes proinflammatory cytokine production from macrophages. *Nat. Commun.* **8,** 14133 doi: 10.1038/ncomms14133 (2017).

**Publisher's note:** Springer Nature remains neutral with regard to jurisdictional claims in published maps and institutional affiliations.

## Supplementary Material

Supplementary Information Supplementary figures.
